# Inflammatory Breast Cancer: The Cytokinome of Post-Mastectomy Wound Fluid Augments Proliferation, Invasion, and Stem Cell Markers

**DOI:** 10.3390/cimb44060187

**Published:** 2022-06-17

**Authors:** Alshaimaa Tarek, Shrouk Khalaf El-Sayed, Wendy A. Woodward, Mohamed El-Shinawi, Jon Mark Hirshon, Mona Mostafa Mohamed

**Affiliations:** 1 Department of Zoology, Faculty of Science, Cairo University, Giza 12613, Egypt; ssunrise268@gmail.com; 2 Maadi Military Hospital, Maadi, Cairo 11711, Egypt; 3 MD Anderson Cancer Center, Welch Inflammatory Breast Cancer Research Program and Clinic, Department of Radiation Oncology, The University of Texas, Houston, TX 77030, USA; wwoodward@mdanderson.org; 4 Department of General Surgery, Faculty of Medicine, Ain Shams University, Cairo 11566, Egypt; mohamedshinawi@gu.edu.eg; 5 Sector of International Cooperation, Galala University, Suez 43511, Egypt; 6 School of Medicine, University of Maryland, Baltimore, MD 21201, USA; jhirshon@umaryland.edu

**Keywords:** inflammatory breast cancer, wound healing fluid, tumor recurrence

## Abstract

Inflammatory breast cancer (IBC) is an aggressive phenotype with a high recurrence and low survival rate. Approximately 90% of local breast cancer recurrences occur adjacent to the same quadrant as the initial cancer, implying that tumor recurrence may be caused by residual cancer cells and/or quiescent cancer stem cells (CSCs) in the tumor. We hypothesized that wound fluid (WF) collected after modified radical mastectomy (MRM) may activate cancer cells and CSCs, promoting epithelial mesenchymal transition (EMT) and invasion. Therefore, we characterized the cytokinome of WF drained from post-MRM cavities of non-IBC and IBC patients. The WF of IBC patients showed a significantly higher expression of various cytokines than in non-IBC patients. In vitro cell culture models of non-IBC and IBC cell lines were grown in media conditioned with and/without WF for 48 h. Afterwards, we assessed cell viability, the expression of CSCs and EMT-specific genes, and tumor invasion. Genes associated with CSCs properties and EMT markers were regulated in cells seeded in media conditioned by WF. IBC-WF exhibited a greater potential for inducing IBC cell invasion than non-IBC cells. The present study demonstrates the role of the post-surgical tumor cavity in IBC recurrence and metastasis.

## 1. Introduction

Breast cancer is associated with a high mortality in women worldwide [1]. Breast cancer treatment is based on several factors, including cancer stage, size, and metastasis [2]. Treatment uses several protocols, such as targeted therapies, hormone therapy, radiation therapy, and surgery. Inflammatory breast cancer (IBC) is the most aggressive phenotype and exhibits a high mortality rate in North African countries characterized by a low response to the mentioned breast cancer therapies [3].

IBC is rare in breast cancers in Western countries; however, IBC is responsible for 10% of breast cancer-related death [4]. Non-IBC shows high survival rates compared to IBC [5]. IBC is characterized by a high invasive potential, the carcinoma cells of IBC can invade dermal lymphatic and lymphatic vessels, forming a cluster of cells or tumor emboli due to high expression of the adhesion molecule E-cadherin, which adhere cells together, forming tumor emboli [6]. IBC metastases to distant organs as clusters of cells [7]. Tumor emboli are a unique feature of IBC [5] and characterized by the overexpression of the cell adhesion molecule E-cadherin and CSC markers, such as CD44(+)/CD24(−) and ALDH1 [8]. These factors increase resistance to chemo- and radiotherapy [9]. CSCs represent a small subpopulation in tumor bulk and demonstrate self-renewal and differentiation properties seen in normal stem cells [10].

Patients with IBC receive treatment that includes neoadjuvant chemotherapy, modified radical mastectomy (MRM), post-mastectomy radiotherapy, and adjuvant therapy, yet survival rates remain poor [11]. Surgery is widely used for breast cancer, and a link between surgery and tumor recurrence is reported [12]. Ninety percent of local breast cancer recurrences occur adjacent to the same quadrant as the primary tumor, suggesting that tumor recurrence may be caused by residual cancer cells and/or quiescent CSCs in the post-MRM tumor bed [13]. MRM causes inflammation and wound healing responses [14].

The process of wound healing promotes the proliferation and activation of immune cells that modulate the release of cytokines, chemokines, and growth factors essential for wound repair and closure [15]. Most cytokines released during inflammation and wound healing, such as IL-6 and IL-8, promote tumorigenesis [16]. Thus, wound fluid might stimulate residual and/or quiescent stem cells in the post-mastectomy tumor bed [12]. CSC stemness in epithelial tissues is promoted via the epithelial to mesenchymal transition (EMT) [17,18,19]. For instance, EMT transcription factors (EMT-TF) inducers, including Twist, Six1 and Snail, promote CSC stemness and tumorigenesis in various mouse models of breast cancer development [20,21,22]. EMT is the transformation of epithelial cells into a mesenchymal spindle shape [21]. Inflammation is an effective inducer of EMT in tumor cells and may induce cancer cells to produce pro-inflammatory factors, such as IL-6, IL-8, and TNF-α [22]. These factors activate stemness that sustain breast cancer progression [23]. Indeed, EMT processes during wound healing encourage the detachment of epithelial cancer cells from the primary site, transition to mesenchyme-like cells, invasion into the bloodstream, and metastasis to secondary sites [24]. The EMT transcription factor, SNAIL (Snail Family Transcriptional Repressor-1) is overexpressed in IBC cell lines compared to non-IBC cell lines [25]. SNAIL is also highly expressed during wound healing and carcinogenesis in colon cancer [26,27]. SNAIL/PDGF-BB expression in tumor microenvironments also promotes EMT, angiogenesis, and ECM remodeling in colon cancer [28]. Despite this, the role of WF after surgery in non-inflammatory breast cancer and in inflammatory breast cancer in general is still unknown.

In this regard, in the present study, we hypothesized that WF collected after MRM may activate residual cancer cells and/or CSCs, promoting EMT and invasion, and initiating tumor recurrence and metastasis.

We characterized the cytokinome of WF drained from the post-MRM cavity of non-IBC and IBC patients using a RayBio™ Human Cytokine Antibody Array-3. The in vitro effects of WF on non-IBC and IBC cell viability, CSCs, EMT gene expression, and cell motility and invasion were examined in vitro. Our results provide a better understanding of how the post-surgical tumor cavity may contribute to IBC metastasis, recurrence, and subsequent high mortality. Future studies are warranted to target candidate cytokines in WF for combined therapy with currently used IBC treatments.

## 2. Materials and Methods

### 2.1. Patient Enrollment

In the present study, ethical approval was obtained from the Institutional Review Board (IRB) approval of Ain-Shams University (IRB#0006379). All patients that participated in the study signed a consent form that approved their participation. Patients were diagnosed by clinical and pathological examination, mammography, ultrasound, and biopsy [29]. The present study recruited 38 hormonal positive receptor estrogen (ER) or progesterone (PR) breast cancer patients with non-IBC (*n* = 24) and IBC (*n* = 14). We excluded patients suffering from viral infections (HCV, HBV, and HIV), autoimmune illnesses, and woman pregnant women or lactating at diagnosis.

### 2.2. Wound Fluid (WF) Preparation

Between 15 and 20 mL of post-mastectomy WF was collected into sterile containers during the first 24 h after surgery. WF was directly collected from wounds in a sterile drainage bag. No additional substances or preservative were added. WF was immediately transferred to the laboratory for analysis. WF was first centrifuged at 1500 rpm for 5 min to separate fluid and pellet. The supernatant was filtered, divided, and stored at −80 °C for later use.

### 2.3. Human Cytokine Antibody Array

The proteomics composition of WF was quantitatively evaluated using RayBio^TM^ array-3 (RayBiotech Life, Peachtree Corners, GA, USA), according to the manufacturer’s recommendations, which detects 42 distinct cytokines, chemokines, and growth factors. In brief, antibody arrays were placed in an incubation tray and incubated for 1 h at room temperature (RT) with gentle shaking in blocking buffer. The membranes were then incubated overnight at 4 °C with 1000 μg protein per array of WF, followed by several washes after incubation. The membranes were treated with a biotinylated antibody cocktail for 2 h at RT with shaking, followed by 2 h incubation with HRP-Streptavidin. Next, membranes were developed using the kit chemiluminescence detection reagent against X-ray CL-XPosure film (Thermo Fisher Scientific, Waltham, MA, USA) and processed using Kodak Developer and Kodak Fixer (Kodak, France). Using ImageJ software (National Institutes of Health, Bethesda, MD, USA), signal intensity values representing identified cytokines, chemokines, and growth factors were subtracted from background staining and adjusted to positive controls on the same membrane as previously described [30]. WF of non-IBC (*n* = 11) and IBC (*n* = 11) patients and signal intensity of identified cytokines are provided as mean ± SEM. A Student’s *t*-test was used to evaluate differences in cytokine/chemokine/growth factor secretion levels between non-IBC and IBC.

### 2.4. Cell Culture

MDA-MB-231, representing the non-IBC cells, and Sum-149, representing the IBC cells, were kindly provided as a gift by Professor B.F. Sloane (Wayne State University, Detroit, 48202, MI, USA). MDA-MB-231 cells were grown in Dulbecco’s modified Eagle’s medium (DMEM), including 10% FBS, 1% glutamine, and 1% penicillin/streptomycin antibiotics (complete medium). Sum149 cells were cultured in Hams F-12 media with 5 g/mL insulin, 1 g/mL hydrocortisone, 1% antibiotics (penicillin/streptomycin), and 5% fetal bovine serum (complete medium). Breast cancer cells were incubated at 37 °C, in a humidified 5% CO_2_ incubator for WF studies. Unless otherwise stated, all tissue culture reagents, media, fetal bovine serum, and supplies were purchased from Lonza (Lonza, Walkersville, MD, USA).

At 80% confluence, MDA-MB-231 and Sum-149 cells were subcultured and seeded in complete media. Then, MDA-MB-231 culture medium was replaced by serum-free medium and non-IBC WF, and Sum-149 cells culture medium was replaced with serum-free medium and IBC WF under the same conditions. The cells were incubated for 48 h. MDA-MB-231 and Sum-149 control cells were seeded in their culture media supplemented with 3% FBS.

### 2.5. MTT Cell Viability Assay

Using MTT [3-(4,5-dimethyl-2- thiazolyl)-2,5-diphenyl-2H-tetrazolium bromide] assay, we studied the effect of WF on MDA-MB-231 and Sum-149 cell viability. Briefly, MDA-MB-231 and Sum-149 cells were seeded in triplicate at a density of 5000 cells/well in 96-well plates as previously described [31]. Different concentrations of WF were prepared in culture media (*V*:*V*) obtaining 1%, 3%, 5%, 10%, 20%, and 25% WF concentrations. MDA-MB-231 cells were grown in medium supplemented with all mentioned concentrations of non-IBC WF, and for control, the culture medium was supplemented with 3% FBS for 48 h. Twenty percent WF was the most appropriate concentration for cell stimulation.

The culture medium for Sum-149 was replaced with serum-free medium supplemented with 20% WF for treated cells and with 3% FBS for control for 48 h. Then, 10 μL of 5 mg/mL MTT was added per well and incubated for 4 h. MTT was removed, and 100 μL dimethyl sulfoxide (DMSO) was added per well to stop the reaction. Absorbance was measured at 570 nm using Infinite^®^ 200 PRO NanoQuant (Tecan Männedorf, Zürich, Switzerland). Data are represented as mean ± SEM. A one-way ANOVA test was used to evaluate significant differences (*p* < 0.05), and Duncan’s test was used to evaluate different concentrations using software IBMSPSS version 25 for windows (SPSS, Chicago, IL, USA).

### 2.6. Invasion Assay

To test whether WF collected from non-IBC and IBC breast cancer patients possess chemotactic properties that induce invasion of MDA-MB-231 and Sum-149 breast cancer cell lines, we utilized BioCoat Matrigel Invasion Chambers with filters coated with reconstituted basement membrane (BD Biosciences, San Jose, CA, USA) [32]. We seeded MDA-MB-231 and Sum-149 cells at a density of 3 × 10^4^ cells per well in serum-free media in the upper chamber after re-hydrating the membrane for 2 h in serum-free media as described in kit manufacturer guidelines. Culture media supplemented with 20% WF from non-IBC and IBC patients were placed in the lower chamber of BioCoat Matrigel Invasion Chambers. For control cells, we added culture media supplemented with 3% FBS in the lower chamber and in the upper chamber we seeded MDA-MB-231 and Sum149 cells. Control experiments ran in parallel with cells seeded with non-IBC and IBC WF in the lower chamber. Invasion chambers were incubated at 37 °C in a CO_2_ humidified atmosphere for 48 h in 5% CO_2_. Next, cells were washed with PBS, fixed with methanol, and stained with 0.2% crystal violet followed by washing the membrane several times with distilled water to remove excess stain. Non-invasive cells that remained attached to the upper side of the membrane were gently removed with a cotton swap, as previously described [33].

Cells that invade the lower side of the membrane due to the chemotactic effect of post-surgical WF were photographed in different fields using light microscopy. Invading cells were counted with ImageJ software (National Institutes of Health, MD, USA). The number of the invading cells was calculated as the mean number of cells in the lower chamber that invaded due to the effect of post-surgical tumor microenvironment fluid, divided by the mean number of cells that had invaded through control membranes, and multiplied by 100 [32].

### 2.7. Quantitative Real-Time PCR Method

The total RNA was isolated from MDA-MB-231 and Sum-149 breast cancer cell using Trizol reagent (Thermo Fisher Scientific, Mississauga, ON, Canada). A High-Capacity cDNA Reverse Transcription Kit (Thermo Fisher Scientific, Mississauga, ON, Canada) was used with 1 µL RNA to obtain complementary DNA (cDNA) [5]. Quantitative real-time PCR was conducted for CD24 (100133941), CD44 (960), E-cadherin (999), and Vimentin (7431) genes using SYBR Green qPCR master mix (Applied Biosystems, Foster, CA, USA) following the manufacturer’s instructions. Reaction included 12.5 µL of SYBR green master mix (Applied Biosystems, Brumath, France), 1 µL of each primer (10 pmol/µL) (Vivantis technologies, SGR, Malaysia) CD24 (forward 5′-CTCCTACCCACGCAGATTTATTC-3′ and reverse 5′-AGAGTGAGACCACGAAGAGAC-3′), CD44 (forward 5′-CTGCCGCTTTGCAGGTGTA-3′ and reverse 5′-CATTGTGGGCAAGGTGCTATT-3′), E-cadherin (forward 5′-ATTTTTCCCTCGACACCCGAT-3′ and reverse 5′-TCCCAGGCGTAGACCAAGA-3′), Vimentin (forward 5′-GACGCCATCAACACCGAGTT-3′ and reverse 5′-CTTTGTCGTTGGTTAGCTGGT-3′), 2.5 µL of cDNA, and 8 µL of RNase-free water for a total reaction volume of 25 µL. Gene expression levels were quantified in a Step One Plus detection system (Applied Biosystems, CA, USA). We calculated expression levels using the 2−ΔΔCt method after normalizing data to 18S ribosomal RNA (rRNA) (Applied Biosystems, CA, USA) (forward 5′-GGATGTAAAGGATGGAAAATACA-3′ and reverse 5′-TCCAGGTCTTCACGGAGCTTGTT-3′).

### 2.8. Bioinformatic Analysis and Construction of a PPI Network

We used the STRING database (Search Tool for the Retrieval of Interacting Genes/Proteins), an online server commonly used to identify interactions between known proteins and predicted proteins, and thus investigated protein–protein interaction (PPIs) between differentially expressed genes (DEGs). The PPI network included all active interaction sources, such as experimental validation, co-expression analysis, literature mining, and records in the database [34]. We constructed a PPI network via DEGs pairs with combined scores >0.4. The network was visualized using Cytoscape version 3.8.2, National Institute of General Medical Science, Bethesda, MD, USA.

## 3. Results

### 3.1. Clinical and Pathological Characterization of Patients

The clinical and pathological characterization of non-IBC (*n* = 24) and IBC (*n* = 14) patients enrolled in the present study is shown in Table 1. Statistical analysis showed that the status of lymph node metastasis was significantly higher (*p* = 0.031) in IBC patients than non-IBC patients. Estrogen and progesterone hormonal receptors were significantly more expressed in non-IBC patients than IBC patients (*p* = 0.003 and 0.001, respectively).

### 3.2. WF Collected from IBC Patients Showed Highly Significant Levels of GRO, IL7, IL-α, and PDGF-BB Cytokines Compared to Non-IBC Patients

**Figure 1:**
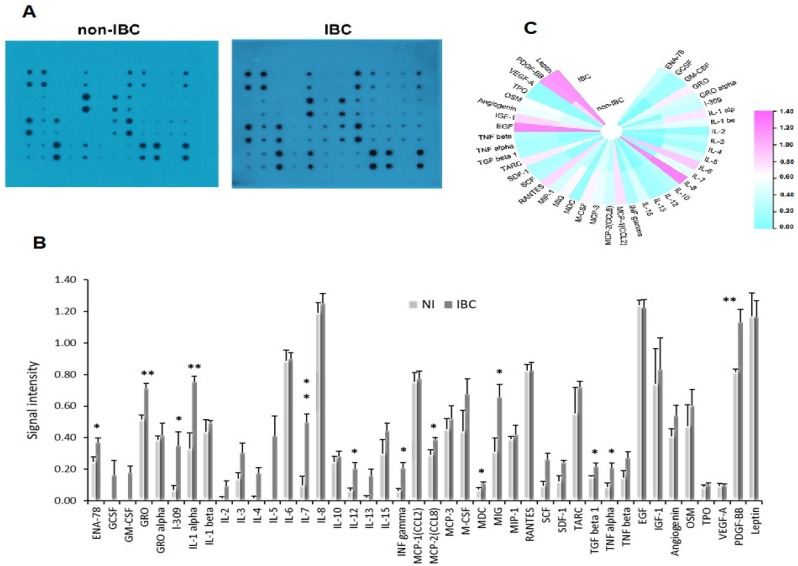
Human cytokine array for WF collected from IBC and non-IBC patients. Cytokine profile of WF collected from non-IBC and IBC breast cancer patients. (**A**) X-rays represent Human Cytokine Antibody Array™, which assessed 42 cytokines, chemokines and growth factors. Dots represent the intensity value of antibodies for specific WF cytokine (antigen). (**B**) Bars indicate the mean ± SEM of the signal intensity of each cytokine released in WF of non-IBC (*n* = 11) and IBC (*n* = 11) measured using ImageJ software. * represents significant *p* ˂ 0.05, ** represents significant *p* ˂ 0.01 as determined by Student’s *t*-test. (**C**) LogFC heatmap of the image data of cytokine array expression of the proteomic composition of wound fluid of both non-IBC and IBC, which revealed 42 distinct cytokines, chemokines, and growth factors; highly expressed cytokines are displayed as shades of pink and minimally expressed cytokines are shown as shades of aqua.

Cytokine profile of non-IBC and IBC WF assessed using RayBio™ human cytokine antibody array-3 showed significantly high levels of 13 cytokines, chemokines, and growth factors in WF for IBC compared to non-IBC patients (Figure 1A,B; Table 2). Heatmap of proteomic composition of non-IBC and IBC wound fluid identified 42 distinct cytokines, chemokines, and growth factors. Highly expressed proteins are displayed in shades of pink, and minimally expressed genes are shown in shades of aqua, (Figure 1C). The most detected cytokines were GRO, IL7, IL-1α, and PDGF-BB.

### 3.3. WF Increases the Viability and Proliferation of Breast Cancer Cell Lines

**Figure 2:**
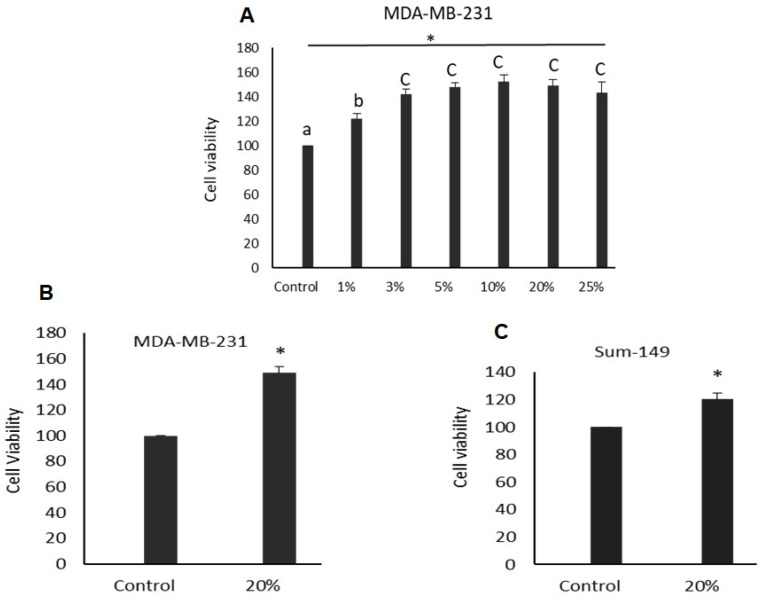
MTT assay. Bars represent cell viability for MDA-MB-231 and Sum-149 breast cancer cell lines cultured in WF of IBC (*n* = 7) and non-IBC (*n* = 12) patients for 48 h assessed by the MTT experiment. (**A**) MDA-MB-231 cells were stimulated for 48 h with various concentrations of WF (1%, 3%, 5%, 10%, 20%, and 25%). * indicates significant difference (*p* < 0.05) as evaluated by one-way ANOVA test. Means with the different letters for different concentrations are significantly different (*p* < 0.05); otherwise, they are not significantly different. (**B**,**C**) MDA-MB-231 and Sum-149 cell viability after being stimulated with 20% WF. The results are based on three independent experiments. Results are shown as mean ± SEM compared to control cell viability; * indicates significant *p* < 0.05 as evaluated by Student’s *t*-test.

We assessed the ability of WF collected from non-IBC and IBC patients to alter the proliferation rate of MDA-MB-231 and Sum-149 breast cancer cell lines. Proliferation was significantly elevated in MDA-MB-231 and Sum-149 cells seeded in media with WF compared to control cells (Figure 2). The proliferation of MDA-MB-231 cells seeded in different concentrations of WF (1%, 3%, 5%, 10%, 20%, and 25%) (Figure 2A) were significantly elevated compared to control cells. Sum-149 cells treated with 20% WF were significantly highly proliferated compared to control cells (*p* < 0.05) (Figure 2C).

### 3.4. WF Fluid of IBC Patients Enhances Invasion of Sum-149 Cells More Than Non-IBC

**Figure 3:**
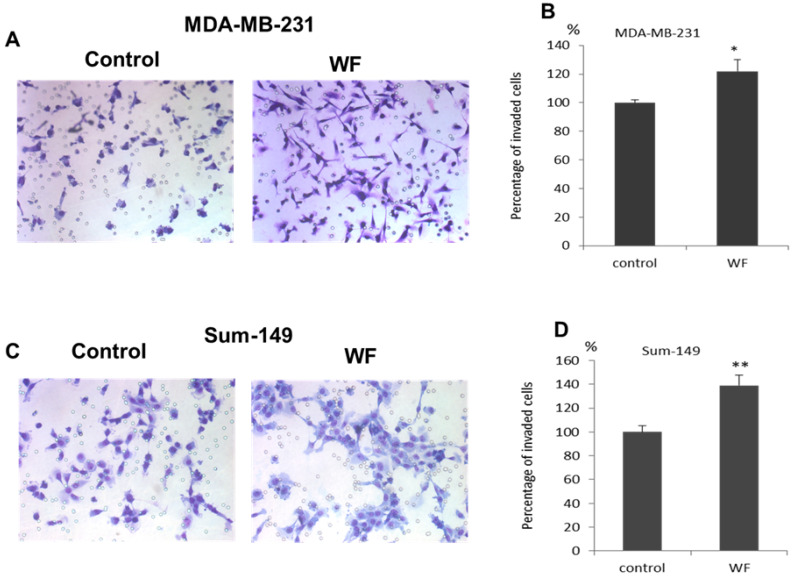
WF induces invasion of MDA-MB-231and Sum149 cells cultured in WF of non-IBC (*n* = 12) and IBC patients (*n* = 12). (**A**,**C**) Images represent the invasiveness of MDA-MB-231 and Sum149 cells through Matrigel-coated filters due to the effect of 20% WF placed in the lower chamber and collected from non-IBC (*n* = 24) and IBC (*n* = 14) for treated cells and control cells. (**B**,**D**) Quantification of invaded cells compared to control cells. Results are expressed as mean ± SEM; ** represents *p* ≤ 0.001 and * *p* < 0.004 as determined by Student’s *t*-test.

For the BD BioCoat™ BD Matrigel™ Invasion Chamber assay, the results show that WF of non-IBC promotes the invasive capabilities of MDA-MB-231 and WF of IBC promotes the Sum-149 cells invasive capabilities. The MDA-MB-231 and Sum-149 cells invade the coated filters of Matrigel invasion chambers due to the chemotactic properties of the cytokines, chemokines, and growth factors that constitute WF with significant differences (122% *p* = 0.024) for MDA-MB-231 cells compared to control cells (without WF) and 139% *p* = 0.001) for Sum-149 cells compared to control cells (without WF) (Figure 3).

### 3.5. WF Alters the Expression of Genes Associated with Stem Cell Properties and EMT

**Figure 4:**
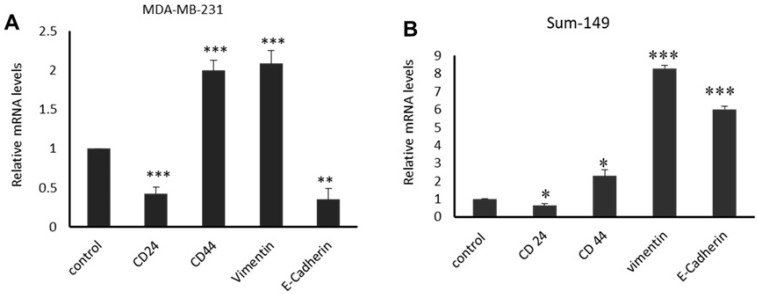
Quantitative real-time PCR. Expression levels of CD24, CD44, vimentin, and E-cadherin for breast cancer cell lines MDA-MB-231 and Sum-149 seeded in WF of non-IBC (*n* = 12) and IBC (*n* = 9) patients. (**A**,**B**) MDA-MB-231 and Sum-149 breast cancer cell lines were seeded for 48 h in culture media supplemented with 3% FBS (control), and 20% WF for treated cells. Bars represent relative quantification of mRNA expression of CD24, CD44, vimentin, and E-cadherin analyzed using 2−ΔΔCt method after normalization to 18S (rRNA). Data represent mean ± SEM and were analyzed using the Student’s *t*-test. * *p* ≤ 0.05, ** *p* ≤ 0.01, and *** *p* ≤ 0.001.

Genes associated with stem cell properties *CD44*, *CD24* and EMT markersm such as E-cadherin and vimentinm were altered when seeded in media conditioned with WF collected from non-IBC and IBC patients (Figure 4). The mRNA expression of CD44 and Vimentin was significantly upregulated (*p* = 0.0007), (*p* = 0.001) compared to the control in the non-IBC cell line for MDA-MB-231 cells seeded in media conditioned by WF and collected from non-IBC patients. Meanwhile, CD24 was significantly downregulated (*p* = 0.0009), in comparison with control, and the expression level of E-cadherin was decreased (*p* = 0.006) in comparison with the control (Figure 4A).

For IBC cell line Sum-149, the level of expression of CD44, vimentin, and E-cadherin were significantly upregulated—(*p* = 0.04), (*p* = 0.000), and (*p* = 0.000), respectively—and seeded in media conditioned by WF collected from IBC patients compared to the control. On the other hand, the mRNA expression of CD24 was significantly downregulated (*p* = 0.04) in comparison with the control (Figure 4B). It should be noted that WF collected from IBC patients induced the expression of E-cadherin by Sum-149. E-cadherin overexpression is hallmark of IBC tumor emboli. The high expression of E-cadherin characterizes IBC carcinoma cells, since IBC invades as clumps of cells attached together.

### 3.6. Bioinformatic Analysis and Construction of PPI Network

**Figure 5:**
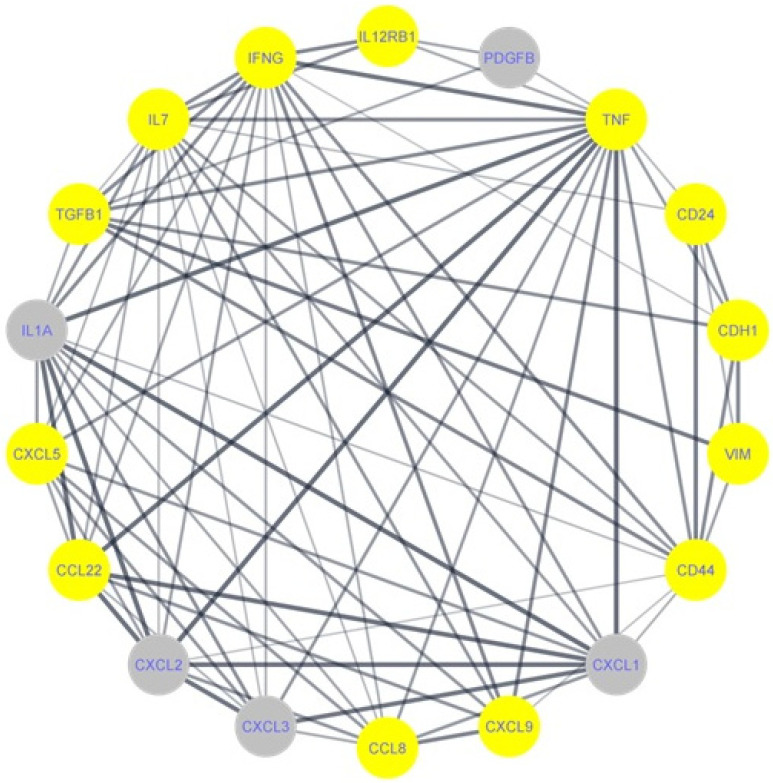
PPI network of WF cytokine array with m-RNA data from the WF-treated cell lines, MDA-MB-231 and Sum-149. Nodes represent all upregulated cytokines in WHF of both non-IBC and IBC cell lines, mRNA of CD24 and CD44 as cancer stem cell markers, and vimentin and E-cadherin as epithelial mesenchymal transition markers. We focused on GRO (CXCL1, CXCL2, CXCL3), IL-1α, IL-7, and PDGF-BB, which are marked with the grey color, and their interaction with biomarkers of stem cell and mesenchymal transition process.

We constructed a PPI network via a STRING database to study the interaction between CD24, CD44 as cancer stem cell markers, and vimentin and E-cadherin as epithelial mesenchymal transition markers with a significant expression of the proteomics composition of WHF. We focused on GRO (CXCL1, CXCL2, CXCL3), IL-1α, IL-7, and PDGF-BB, which are shown in the PPI network as grey color nodes, and their interactions with biomarkers of stem cells and the mesenchymal transition process (Figure 5).

Additionally, STRING database gave us a functional enrichment analysis of all involved gene sets according to the KEGG Database, in order to determine the higher scores of pathways of these gene sets. The PPI gene set was found to be enriched in different carcinogenic pathways, including in cytokine–cytokine receptor interactions, the JAK-STAT signaling pathway, pathways in cancer, the TNF signaling pathway, chemokine signaling pathway, EGFR tyrosine kinase inhibitor resistance, proteoglycans in cancer, TGF-beta signaling pathway and microRNAs.

We searched the Tissue Expression Database to find support for our PPI result. The database showed human genes associated with breast cancer stem cells based on text mining Z score. The highest z-scores were CD24 and CD44; Table 3. Additionally, the Tissue Expression Database showed that extracellular matrix-associated cytokines, such as IL-1α, which was also involved in our PPI, were found to induce the expression of the stem cell antigen CD44. The pro-inflammatory cytokines IL-1α and IL-1β, as well as the interleukin-1 receptor antagonist (IL-1rα), are the most prominent members, all of which are involved in the onset and progression of inflammatory processes promoting carcinogenesis.

## 4. Discussion

Breast cancer recurrences still lead to low survival rates despite breakthroughs in treatment [35]. Recurrences at the primary tumor site are common, regardless of precautions taken after surgery and microscopic screenings to ensure that safety margins are clear of residual cancer cells [36]. Surgery alters the tumor bed by triggering inflammatory responses. For instance, several cytokines and growth factors are produced during inflammatory and wound healing processes following surgery. These regulatory factors might activate residual tumor cells and/or dormant stem cells [12]. Thus, surgery and tumor recurrence may be linked [13]. Post-MRM wound fluid is rich in cytokines and growth factors that are essential for the wound healing process. These factors may also encourage inflammation, which activates cancer cells and/or quiescent cancer stem cells. Our current data are consistent with these findings. The cytokinome of WF collected from IBC patients exhibited 13 cytokines, chemokines, and growth factors (Figure 2 heat map; Table 2). Growth-regulated oncogene (GRO), IL-1α, IL-7, and PDGF-BB were highly detected in the WF collected from IBC compared to non-IBC. Chemokine GRO, including isoforms GRO-α, GRO-β, and GRO-γ, plays a key role in wound healing by controlling cell migration and angiogenesis [16]. IBC cells and IBC-activated monocytes/macrophages release large quantities of GRO chemokines, which activate the JAK/STAT3 pathway and promote mesenchymal and CSC-like phenotypes [25].

IL-1α is a member of IL-1 family and functions as a pro-inflammatory cytokine that promotes the expression of other cytokines, such as IL-6 [37]. M. Dúcka and colleagues showed that overexpression of IL-1α and the accumulation of its recombinant protein stimulated NF-κB signaling and restored the expression of hallmark inflammatory genes repressed by c-Myb [38]. HER2 increased drug-resistance-related CSCs in HER2+ breast cancer patients, yet the blocking of IL-1α signaling improved chemotherapy efficacy when combined with cisplatin and paclitaxel. Such results reflect the importance of combined therapy in breast cancer patients due to the interference of cytokines with targeted therapy [39], as we previously showed [40].

The aberrant expression of IL-7 and IL-7Ra polymorphism is an interesting marker for breast carcinogenesis based on molecular subtypes [41]. Furthermore, IL-7 expressed by cancer-associated fibroblasts stimulated the stemness and tumor growth of breast cancer [42]. IL-7-expressing cancer-associated fibroblasts (CAFs) promoted breast tumor growth and provided critical niches for the maintenance of breast cancer stemness. SDF-1 is an important niche factor in IL-7-expressing CAFs, indicating that the CXCL12 (SDF-1)/CXCR4 pathway may be a useful anti-cancer stem cell therapeutic (anti-CSC) target [42]. PDGF-BB is one of the factors that has a significant impact on the survival rate in ER+ breast tumors [43]. PDGFR inhibitors (ponatinib or sunitinib) are used to block PDGF-BB signaling in combination with tamoxifen. This combination inhibited cell growth and decreased the expression of matrix metalloproteinase-1 (MMP-1), a gene associated with metastasis.

We assessed the ability of post-MRM WF to alter the proliferation, invasion, and stem cell markers of non-IBC and IBC cancer cells. Firstly, we studied whether WF could alter the proliferation of breast cancer cell lines. The results show that breast cancer cells seeded in media conditioned by WF induce their proliferation rate. It was reported that post-MRM WF induced the proliferation of breast cancer cell, which is in agreement with our results [35].

Furthermore, qRT-PCR showed that WF significantly increased the gene expression of CD44 in both non-IBC and IBC patients compared to control cells. Additionally, we found that the gene expression of CD24 was significantly downregulated in IBC and non-IBC cells. Previously, tumor cells with *CD44high/CD24low*, and diagnosed with invasive ductal carcinoma, showed a poor overall survival rate after treatment [44]. Additionally, there is a link between increased CD44+/CD24− and the expression of the SLUG EMT marker [45]. Therefore, for E-cadherin and Vimentin, epithelial and mesenchymal markers expressions in MDA-MB-231 and Sum-149 cells incubated in WF were examined at gene level. We observed that the mRNA expression of the epithelial marker, E-cadherin, was significantly overexpressed in Sum-149 cells compared to the decrease observed in MDA-MB-231 cells. This result is in agreement with a previous study, which showed that E-cadherin is overexpressed in IBC compared to non-IBC patients [6]. In IBC, E-cadherin plays a different role than in non-IBC patients. The loss of E-cadherin expression in non-IBC is linked to increased tumor proliferation and metastasis, as well as a poor prognosis [46]. On the other hand, increased E-cadherin in IBC allows cancer cells to migrate as clumps of cells within lymphatic and blood vessels, resulting in distant metastasis and multiorgan failure in IBC patients, which has been linked to disease aggressiveness and a lower survival rate [47].

Additionally, the expression of Vimentin, a mesenchymal marker, was significantly upregulated in Sum-149 and MDA-MB-231 cells compared to control cells. Similar to this finding, Yamashita et al. [48] showed that aggressive phenotypes and a poor prognosis in breast cancer patients was attributed to the upregulation of Vimentin. Since we assessed that cells treated with WF might induce EMT; we used an in vitro invasion assay to confirm EMT as the epithelial switch to a more mesenchymal phenotype. WF from non-IBC and IBC patients enhanced the invasion of Sum-149 cells more than MDA-MB-231. The present results agree with other study, which showed that MCF-7 and MDA-MB-468 cells in a 3D culture invaded much faster following stimulation with WF, agreeing with our findings [49]. We can conclude that the cytokinome of WF collected within 24 h after MRM contains cytokines, chemokines and growth factors crucial for the growth of residual cancer cells and/or cancer cells. This activity promotes tumor recurrence and spreads to distant organs. Future studies are essential to elucidate detailed mechanisms of post-MRM in IBC recurrence and metastasis.

## 5. Conclusions

In conclusion, WF promotes the proliferation, EMT markers (E-cadherin and Vimentin), and CSC markers (*CD44high/CD24low*) of MDAMB-231 and Sum-149 breast cancer cells. Furthermore, WF increases the cell invasion of IBC more than non-IBC. These findings improve our understanding of post MRM WF in encouraging IBC recurrence, invasion, and metastasis. Future studies are warranted to identify therapeutic target candidates present in WF.

## Institutional Review Board and Informed Consent

Ethical approval was obtained from Institutional Review Board (IRB) approval of Ain-Shams University (IRB#0006379). All patients involved in this study signed a consent form approving their participation in the study.

## Figures and Tables

**Table 1:** Clinical and pathological characterization of non-IBC versus IBC patients.

Characteristic	Non-IBC (*n* = 24)	IBC (*n* = 14)	*p* Value
**Age (year)**			
Range	29–80	36–69	0.73 ^a^
Mean ± SD	50.17 ± 12.41	51.61 ± 11.54	
NA	0	1	
**Tumor size (cm)**			
Mean ± SD	3.89 ± 2.62	5.95 ± 3.74	0.710 ^b^
≤4	11 (52.4%)	5 (45.5%)	
>4	10 (47.6%)	6 (54.5%)	
NA	3	3	
**Tumor grade**			
G1	0 (0%)	1 (7.7%)	0.247 ^b^
G2	18 (78.3%)	11 (84.6%)	
G3	5 (21.7%)	1 (7.7%)	
NA	1	1	
**Axillary lymph node metastasis**			
≤4	14 (63.6%)	3 (25.0%)	0.031 *^b^
>4	8 (36.6%)	9 (75.0%)	
NA	2	2	
**Lymphvascular invasion**			
Negative	10 (45.5%)	3 (21.4%)	0.143 ^b^
Positive	12 (54.5%)	11 (78.6%)	
NA	2	0	
**ER**			
Negative	1 (4.2%)	6 (42.9%)	0.003 *^b^
Positive	23 (95.8%)	8 (57.1%)	
NA	0	0	
**PR**			
Negative	1 (4.2%)	7 (50.0%)	0.001 *^b^
Positive	23 (95.8%)	7 (50.0%)	
NA	0	0	
**Her-2**			
Negative	20 (83.3%)	8 (57.1%)	0.077 ^b^
Positive	4 (16.7%)	6 (42.9%)	
NA	0	0	

Data are reported as mean ± SD. ^a^ Student’s *t*-test. ^b^ Chi-square test. * Significant *p* value (*p* < 0.05). NA = not available.

**Table 2:** Significant cytokines, chemokines, and growth factors.

Cytokines, Chemokines, Growth Factors	*p* Value
ENA-78	*p* = 0.035
GRO	*p* = 0.001
l-309	*p* = 0.018
IL-1α	*p* = 0.005
IL-7	*p* = 0.001
IL-12	*p* = 0.014
INF-γ	*p* = 0.01
MCP-2	*p* = 0.025
MDC	*p* = 0.034
MIG	*p* = 0.024
TGF beta 1	*p* = 0.027
TNF alpha	*p* = 0.014
PDGF-BB	*p* = 0.007

**Table 3:** Stem cell genes with highest Z-score.

Name	Z-Score	Confidence
**CD24**	6.7	★★★★☆
**CD44**	6.2	★★★★☆
**ALDH1A1**	6.0	★★★☆☆
**ERBB2**	5.4	★★★☆☆
**NANOG**	5.1	★★★☆☆
**NOTCH1**	5.0	★★★☆☆
**POU5F1**	5.0	★★★☆☆
**ESR1**	4.9	★★★☆☆
**SOX2**	4.9	★★★☆☆
**PROM1**	4.9	★★★☆☆
**CTNNB1**	4.9	★★★☆☆
**CDH1**	4.9	★★★☆☆
**SNAI1**	4.8	★★★☆☆
**ZEB1**	4.8	★★★☆☆
**BMI1**	4.7	★★★☆☆
**hsa-miR-200c-3p**	4.7	★★★☆☆
**ENSP00000473391**	4.7	★★★☆☆
**ITGA6**	4.6	★★★☆☆
**STAT3**	4.6	★★★☆☆
**SNAI2**	4.6	★★★☆☆

## Data Availability

The supplied materials, data, and protocols have no restrictions on their availability.
